# Response Attenuation of a Structure Equipped with ATMD under Seismic Excitations Using Methods of Online Simple Adaptive Controller and Online Adaptive Type-2 Neural-Fuzzy Controller

**DOI:** 10.1155/2022/5832043

**Published:** 2022-07-01

**Authors:** Rasoul Sabetahd, Seyed Arash Mousavi Ghasemi, Ramin Vafaei Poursorkhabi, Ardashir Mohammadzadeh, Yousef Zandi

**Affiliations:** ^1^Department of Civil Engineering, Tabriz Branch, Islamic Azad University, Tabriz, Iran; ^2^Multidisciplinary Center for Infrastructure Engineering, Shenyang University of Technology, Shenyang 110870, China

## Abstract

The present study aims to design a robust adaptive controller employed in the active tuned mass damper (ATMD) system to overcome undesirable vibrations in multistory buildings under seismic excitations. We propose a novel adaptive type-2 neural-fuzzy controller (AT2NF). All system parameters are taken as unknowns. The MLP neural network is used to extract the Jacobian and estimate the structural model; then, the estimated model is applied to the controller online. To tune the control force applied to the ATMD and achieve the control targets, the controller parameters are adaptively trained using the extended Kalman Filter (EKF) and the error back-propagation algorithm. A PID controller is also included in this method to increase the stability and robustness of the adaptive type-2 neural-fuzzy controller against seismic vibrations. An online simple adaptive controller (OSAC) is studied to demonstrate the suggested controller's superiority. The OSAC is based on adaptive control of the implicit reference model. In this proposed method, the EKF is used to tune the controller parameters online as a novel feature. The uncertainty associated with identifying the mechanical properties of structures, such as mass and stiffness, is one of the primary challenges in the real-time control of structures. This paper investigates how both controllers cope with parametric uncertainties under far-field and near-field seismic excitation. According to numerical results, the AT2NF controller outperforms OSAC in minimizing the dynamic responses of the structure during an earthquake and accomplishing control objectives when the structure's characteristics change.

## 1. Introduction

Nowadays, some natural disasters, such as large-scale earthquakes and strong winds, can cause significant damage to human life and have injuries and enormous economic consequences [1–17]. As a result, structural control is a modern and efficient scheme for reducing dynamic responses and preventing excessive damage or collapse in structures [18]. Control strategies can ensure an acceptable level of comfort and serviceability to users while allowing the designer to increase flexibility in the structural system and reduce the use of materials [19]. Various control systems have been developed to ensure safety even in excessive vibration in an earthquake or wind excitation. These systems are classified into four general groups, including passive control systems [20, 21], semi-active control systems [22], active control systems [23–25], and hybrid control systems [26, 27]. One of the advantages of the active control system of ATMD over passive control systems such as TMD is its remarkable adaptability and performance for various excitation frequencies. This system is also efficient for transitive vibrations and effectively minimizes the responses resulting from strong earthquakes [28]. The tuned mass damper (TMD) is a typical example of a passive control system with several inherent limitations, such as restriction to a narrow frequency band and sensitivity to parameter adjustment. Notably, TMDs are adjusted to only a specific structural frequency.

On the other hand, buildings subjected to strong earthquakes or wind undergo considerable structural damage, leading to a change in the dominant frequency of the structure consequently. Since the optimal parameters of the TMD damper are a function of the dominant frequency of the main system, the high sensitivity of that to the parameters of the structural model or neglect of the soil-structure interaction may cause an undesirable shock absorption effect [29–32]. Thus, the researchers suggested utilizing several TMDs with different properties to tackle these drawbacks [33–42]. Besides, presenting the nonlinear stiffness or nonlinear damping to linear TMD can be useful for broadening the damping frequency band. For this purpose, the performance of particle tuned mass damper (PTMD) under the complex dynamic loads was examined by Lu et al. in 2009 [40]. The fundamental interaction mechanics consist of energy loss and momentum variation.

The authors exploited the concept of the effective momentum exchange and particle damping technology [43, 44] to propose a particle TMD for dealing with such dynamic loads. This concept specifies the effect of momentum quantity on the performance of the low volumetric filling ratio particle damping. Compared to the conventional ones, the overall stability of this type of TMD is remarkable since it can cover a vast range of damping frequency bands and control the structure response if it is tuned well. Notably, the semi-active and hybrid control strategies are more feasible in implementation than the others and are always based on active control algorithms [23, 28, 36, 45]. The semi-active and hybrid control strategies have remarkable benefits, which make them extremely popular for control purposes. To mention a few, since the semi-active control systems are actually the passive control systems that can tune and change the system's physical properties, they are known as the controllable passive devices. Such semiactive control systems are inherently nonlinear and have many of the advantages of active control systems without the need for a large power source. Also, the control forces in most semi-active control systems are applied in the opposite direction of the structure; thus, the structure's overall stability is maintained. The hybrid controllers are a combination of an active control system and a passive control system. The former is used as a supplementary and improvement of the passive control system's efficiency, and the latter is employed to reduce the required energy in the active control system. The hybrid control strategy can significantly reduce the limitations of the active control systems and has a better performance. Besides, this controller does not lose its good performance even when the energy source faces a problem.

Nevertheless, an active tuned mass damper can be a helpful alternative. The system can work with multiple vibration modes and is considered a feasible option for multi-degree-of-freedom buildings [46]. One of the essential advantages of ATMDs is that a relatively small mass can reduce the response of structures and create high performance [47]. In addition, the active control force employed to transmit this small mass strikingly exerts a secondary inertial force against the vibrations [48]. However, regarding the mass of the entire system, including the building and the mass of the adjustable damper, the driving force requirements applied to the ATMD systems may reach a high level which this situation is unfavorable for all control systems. Since the performance of control systems depends a lot on the control algorithm employed for tuning the control force, the various control algorithms have received much attention as an efficient approach to enhance the performance of an ATMD control system. In other words, they are applied to reduce dynamic vibrations of buildings and enhance the civil engineering structures' capabilities, such as safety and efficient performance against severe environmental excitations. Using an effective control scheme, an appropriate trade-off is created between these contradictory aims, including control force reduction and structural response reduction [49, 50].

The main algorithms widely employed to tune the control force applied to ATMD are LQR [51–53], H2 and H∞ [54, 55], bang-bang control [56], fuzzy logic controller [38, 48, 57, 58], acceleration feedback regulators [59, 60], feedforward and feedback optimal tracking controllers (FFOTC) [61], sliding mode control (SMC) [62–64], fuzzy PID controller [65, 66], and PID controller [53, 67, 68]. Most of the studies in structural control are conducted based on their nominal parameters. However, in real structures, the structural system responses are inevitably uncertain due to the simplification of engineering structures model, estimates, assumptions, and environmental loads that are changeable and unpredictable. On the other hand, the possibility of damage to the structural component due to extreme environmental events like earthquakes and wind loads exists. This situation leads to a change in the structure's characteristics, such as stiffness, natural frequencies, and mode shapes, and subsequently, it creates the characteristics of a time-varying structural system. Therefore, such uncertainties can affect the performance of the control algorithm and probably make it unstable. Also, traditional control methods in this situation do not provide stability and strength required to effectively reduce the structural response resulting from unrecognizable and different external dynamic loading conditions [69–71].

As a result, developing a control scheme that is insensitive to parametric changes in the structural system and is sufficiently robust while providing satisfactory performance for a control system is essential for successful operation. It is noteworthy that the simultaneous use of vibration control and structural health monitoring strategies is necessary to make structures smart [72–74]. Therefore, the structural characteristics of the intelligent structural system must be measured during extreme excitations in real-time. Then, the appropriate control forces must then be applied by the control system to reduce dynamic responses and compensate for possible damage to the structure [62, 75–78].

The linear quadratic regulator (LQR) that is extensively employed for controlling the structural vibrations is considered one of the significant accomplishments of modern control in linear analysis of the systems. According to the optimal quality of LQR, when it is appropriately designed, the control strategy can minimize the cost and response very successfully. Nevertheless, where all cases are unavailable for complete feedback, an observer needs to reconstruct them from the system output. After performing this process for extensive systems, many sensors are required to estimate the observation and quality of state reconstruction. It can significantly increase the cost of the optimal control approach and complicate its implementation. It is also required the controller LQR access to structural parameters like mass, stiffness, and damping. These parameters are not available in many existing structures. Therefore, implementing a control approach that is cost-effective, reliable, predictable, and effective may lead to the calculation of this control solution in the design. Finally, a flexible and secure structure will be created. The adaptive control strategy is a good idea to overcome many uncertainties related to predicting structural system parameters and seismic loads. Since this adaptive strategy can calculate the control gain based on real-time sensed responses, it guarantees desirable performance in the presence of uncertainties [19]. Concerning the various control strategies, significant improvements in adaptive control theory have been made for identifying [79, 80] and controlling linear and nonlinear systems, such as backstepping control [81], model reference control [71, 82], robust adaptive control [83, 84], and indirect adaptive control [85–87].

Notably, recent years have seen a growing interest in adaptive control strategies in structures. For instance, a multi-degree-of-freedom (MDOF) structure in 2000 was controlled through an integrated procedure by Gattulli and Romeo [88]. The numerical analysis of this study was performed through a sliding mode control and model reference adaptive control (MRAC), and acceptable results were obtained. Likewise, a real-time model reference adaptive identification approach was presented by Chu (2009) to exploit online system identification in the MRAC algorithm, considering Lyapunov's direct method in parameter estimation [89]. In a significant advance in 2013, Bitaraf and Hurlebaus employed semi-active adaptive control to examine the seismic excitations in the 20-story tall building with nonlinear behavior and magnetorheological (MR) dampers and finally obtained acceptable results. In 2014, an active control system equipped with an active mass damper was presented by Tu et al. [90]. Model reference adaptive control (MRAC) was utilized in this system, whose parameters' exact values do not need to be calculated.

Regarding adaptive backstepping control, Amini and Ghaderi [91] developed a design procedure for specifying the desired control force for a structure subjected to an earthquake. In this method, the controlled structure's mass, stiffness, and damping matrices were unknown. In a groundbreaking paper from 2018 regarding the online self-tuning approach, Hosseini and Taghikhany exploited a fuzzy inference system to adjust the structural system parameters and improve the defects of the simple adaptive control (SAC) method [92]. The proposed control structure was assessed according to the uncertainty in the model and regardless of that. According to Luyu Li et al., a proposed model reference sliding model control (MRSMC) could minimize the structure's vibration under earthquakes by considering an AMD in a multi-degree-of-freedom (MDOF) nonlinear structure [93]. Mamat et al. proposed a nonsingular terminal sliding mode control to examine the control performance subjected to seismic excitations [62]. Sourni et al. in 2020 introduced an adaptive optimal controller to identify parametric uncertainties in the seismic motion control of structures [94].

Many existing studies in the broader literature [76, 95–101] have successfully implemented adaptive control schemes on structures. Some controllers, such as the Simple Adaptive Controller (SAC), recently studied by Soares et al. [18, 102], can be used to reduce the seismic response of structures under seismic excitations. There is no research in the existing literature on the reduction of seismic responses in an ATMD-equipped structure using an adaptive type-2 neural-fuzzy controller. Since neural networks can approximate any nonlinear function with the desired accuracy and have robust adaptive, self-learning, and self-organization modes under specific conditions, their remarkable practicality is undeniable [83, 103–105]. Therefore, this study presents a novel adaptive type-2 neural-fuzzy network (AT2FN) controller to regulate the active tuned mass damper (ATMD) control force on the 11-story structure. This controller aims to minimize the dynamic responses in the system under near-field and far-fault field excitations, which can cause more uncertainties than type-1 fuzzy controllers and be less sensitive to these parameters without considering the dynamics of the structural system and information related to seismic input excitations.

The multilayer perceptron neural network (MLP) structure is utilized to extract the Jacobian of the system. The online estimation model is then applied to the controller. By combining the extended Kalman filter (EKF) with error back-propagation, the controller parameters used to adjust the ATMD control forces are trained. In this case, the control error signal that is the same as the roof displacement is minimized. In addition, a proportional-integral-derivative controller (PID) is added to the adaptive type-2 neural-fuzzy controller to increase the system's stability and robustness against seismic vibrations. To evaluate the robustness and efficiency of the controllers under different real earthquakes, the performance of the suggested control method is compared with the other control methods that have been presented so far. In addition, the comparison is made between the performance of the AT2NF controller and an online simple adaptive controller (OSAC), which is based on the implicit reference model adaptive control. The OSAC does not require complete identification of the controlled system parameters for obtaining the control gains needed to track the desired behavior in the control process. The main innovation of this controller is that the EKF is used in OSAC for tuning its parameters online. The comparison results imply the remarkable capabilities of the AT2NF controller in meeting the control purposes.

The significant advantages of the proposed control approach are summarized as follows:

- The parameters of the structural system are assumed to be unknown, and the controller is designed in online mode.
- Because the system's dynamics are uncertain, the MLP neural network is used to model and extract the system's Jacobian.
- Type-2 neural-fuzzy controller based on EKF is introduced to enhance the performance of the structural system and reduce the responses caused by various earthquakes.
- Due to the adaptability of this controller, it does not require initial settings by the operator.
- The proposed controller can overcome uncertain parameters and a time-varying system.

## 2. Literature Review

In addition to the related works mentioned in the introduction, many studies have been conducted regarding optimal controllers that were significantly accurate. However, regardless of the benefits of OSAC and AT2NF controllers, the efficiency of the proposed controllers is not on par with the ones presented in this paper. The well-known studies that have successfully designed optimal controllers are mentioned in the introduction comprehensively. Nevertheless, the gap in the literature can be easily observed in Table 1, in which the related works are categorized. Notably, the studies highlighted in Table 1 are novel and limited to 2021 and 2022.

As shown in Table 1, the control methods proposed in the previous studies vary significantly and have brought remarkable accuracy. However, the authors claimed that the OSAC and AT2NF controllers proposed in this study outperform the whole state of art controllers presented so far.

## 3. The Main Relations of Motion Equation in the Structural Model

**Figure 1:**
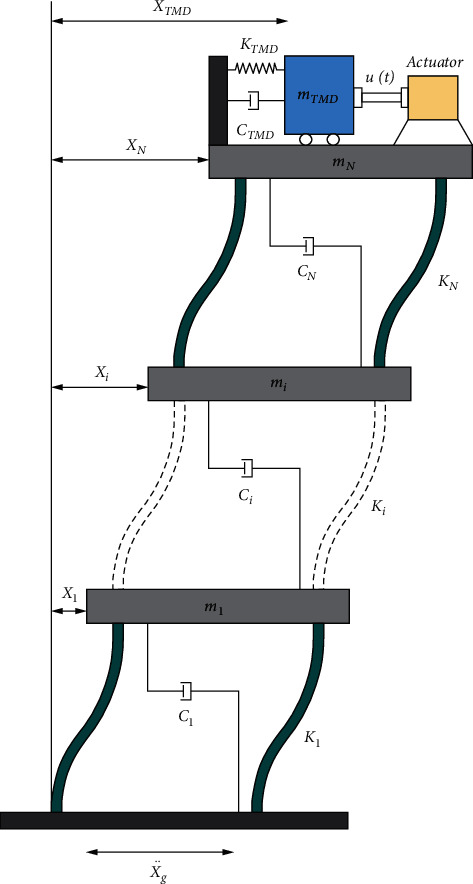
The structural model with ATMD on the top story.

Mass Dampers (MD) are extensively applied to the buildings under external forces like earthquakes to obtain the desired reduction. The passive form of MD is a Tuned Mass Damper (TMD) in which a moving mass is attached to a spring and a viscous damper. In an Active Mass Damper (AMD), an actuator is also connected with the moving mass. The developed form of TMD, namely ATMD, consists of inertial mass, stiffness element, and damping element. The actuator is used to provide the control forces needed to minimize vibrations through an active mass. This section gives the necessary information regarding the motion equations of the structural model considered in this study.  Figure 1 illustrates the structural model equipped with ATMD on the top story, based on the dynamic equation of motion in the N-degree-of-freedom (DOF) shear building. This equation is given below:

(1) $$\begin{array}{c} M\ddot{x}\left(t\right)+C\overset{˙}{x}\left(t\right)+Kx\left(t\right)=-M\Lambda {\ddot{x}}_{g}\left(t\right). \end{array}$$

According to equation (1), *x*(*t*) represents the vectors of structural displacement, $\dot{x}\left(t\right)$ denotes the velocity, $\ddot{x}\left(t\right)$ is the acceleration, and ${\ddot{x}}_{g}\left(t\right)$ represents the excitation acceleration vector for the building with dimensions (*N*+1) × 1, where *N* is the freedom degrees of building. *M*, *C*, and *K* imply (*N*+1) × (*N*+1) mass, damping, and stiffness matrices, respectively, of the structure equipped with an ATMD. Besides, equation (2) shows the (*N*+1) × 1 relative displacement vector, denoted by *X*(*t*).

(2) $$\begin{array}{c} X\left(t\right)={\left[x_{1}\left(t\right),x_{2}\left(t\right),\ldots ,x_{i}\left(t\right),\ldots ,x_{N}\left(t\right),x_{TM\;D}\left(t\right)\right]}^{T}. \end{array}$$

Regarding equation (2), *x*_*i*_(*i* = 1,2,…, *N*) represents the displacement of the *i*-th story comparative to the ground. Accordingly, the expression *x*_*TM* *D*_ indicates the displacement amount between the TMD and the ground. It is noteworthy that the location vector of the excitation acceleration is captured by *Λ*, showing the (*N* + 1) × 1 vector in this case. It is assumed that the masses are cumulated at floor levels; thus, the mass matrix is considered as follows:

(3) $$\begin{array}{c} M=di\;ag\left(m_{1},m_{2},\ldots ,m_{i},\ldots ,m_{N},m_{TM\;D}\right), \end{array}$$

where *m*_*i*_(*i* = 1,2,…, *N*) denotes the mass of the *i*-th story and *m*_*TM*  *D*_ represents the mass of the TMD. One item is added to the degree of freedom in this structure by adding a TMD to the primary structure. Equation (4) indicates the structural stiffness matrix:

(4) $$\begin{array}{c} K=\left[\begin{array}{ccc} \left(k_{1}+k_{2}\right) & -k_{2} & 0\\ -k_{2} & \left(k_{2}+k_{3}\right) & -k_{3}\\ -k_{N} & - & -\\ \vdots & \vdots & \vdots \\ sym & \left(k_{N}+k_{TM\;D}\right) & -k_{TM\;D}\\ \; & -k_{TM\;D} & -k_{TM\;D} \end{array}\right], \end{array}$$

where *k*_*i*_(*i* = 1,2,…, *N*) shows the stiffness of the i-th story, and the stiffness coefficient of the TMD is denoted by *k*_*TM* *D*_. Based on Rayleigh's approach, it is presumed that there is a proportion between the structural damping matrix *C* and the mass and stiffness matrices, as indicated in equation (5). *ω*_1_ and *ω*_2_ in this equation shows the natural structural frequencies in the first and second modes. Furthermore, in the first two modes, *ξ* denotes the structural critical damping ratio.

(5) $$\begin{array}{c} C=\frac{2\xi \omega_{1}\omega_{2}}{\omega_{1}+\omega_{2}}M+\frac{2\xi }{\omega_{1}+\omega_{2}}K. \end{array}$$

The top story of the building is designed to have an active control system. The actuator is located between the structure and the TMD system. The actuator applies the controlled force *u*(*t*) in real-time to the ATMD. Subsequently, its reaction is used in the top story. According to the earlier explanations, the structure movement containing an ATMD is defined by equation (6).

(6) $$\begin{array}{c} M\ddot{x}\left(t\right)+C\overset{˙}{x}\left(t\right)+Kx\left(t\right)=-M\Lambda {\ddot{x}}_{g}\left(t\right)+Du\left(t\right). \end{array}$$

As the control force location vector *D* depends on the location of actuators in the structure, when ATMD is located on the top story, *D* = [0,…, 0, −1, 1]^*T*^ represents the (*N* + 1) × 1 location vector of the control force. In equation (7), the state space form is considered for the dynamic equation of motion in the structural system, for which vector *Z*(*t*) is chosen, as in the equation below:

(7) $$\begin{array}{c} Z\left(t\right)=\left\{\begin{array}{c} x\left(t\right)\\ \dot{x}\left(t\right) \end{array}\right\}. \end{array}$$

Moreover, equation (8) indicates the state matrix *A*, input matrix *B*, vector *H*, and output matrix *G*:

(8) $$\begin{array}{c} A=\left[\begin{array}{cc} 0 & I\\ -M^{-1}K & -M^{-1}C \end{array}\right],\\ B=\left[\begin{array}{c} 0\\ M^{-1}D \end{array}\right],\\ H=\left[\begin{array}{c} 0\\ -\Lambda \end{array}\right],\\ G=\left[\begin{array}{cc} I & 0 \end{array}\right],\\ y\left(t\right)=GZ\left(t\right), \end{array}$$

In this equation, *y*(*t*) denotes the output vector, and *I* and 0 are the identities and zero matrices, respectively, with suitable dimensions. It is noteworthy that element *B* specifies the locations of the control forces. According to the modeling errors, the physical parameters of the nominal and actual structures are different. Since the elements of matrices *M, C*, and *K* in equation (1) are not known exactly, it can be assumed that their values are within certain known intervals, as illustrated in Equation (9).

(9) $$\begin{array}{c} M=\left(1+\Delta_{M}\right)\bar{M},\\ C=\left(1+\Delta_{C}\right)\bar{C},\\ K=\left(1+\Delta_{K}\right)\bar{K}, \end{array}$$

where $\bar{M},\bar{C}$, and $\bar{K}$ denote the nominal values of *M*, *C*, *K*, respectively. Besides, Δ_*M*_, Δ_*C*_, and Δ_*K*_ indicate the uncertainty percent of the structural model.

## 4. The Proposed Control Scheme

The control strategy proposed in this paper is explained in this section. Also, the required information in this regard is presented in detail.

### 4.1. Neural Network Structure of the MLP

**Figure 2:**
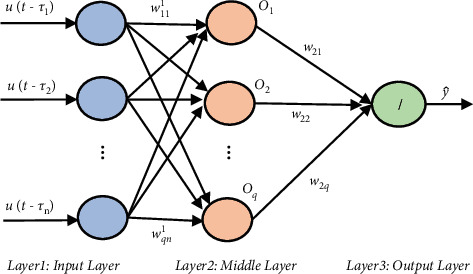
The MLP neural network structure for the system identification [118].

Multilayer feedforward neural networks are among the most important artificial neural network structures (ANN).  Figure 2 illustrates the structure of this neural network employed for the adaptive calculation of the system identification.

The expressions shown in Figure 2 are defined as follows:

- The expression *u*(*t* − *τ*_1_), *u*(*t* − *τ*_2_) …, *u*(*t* − *τ*_*n*_) denotes the inputs of neural networks in which *τ*_1_, *τ*_2_,…, *τ*_*n*_ are constant delays. The MLP neural network receives the previous time samples and the control signal as inputs.
- *u*(*t*) is the sum of the control signal and output system at the instant *t*.
- *w*_11_^1^, *w*_12_^1^,…, *w*_1*n*_^1^ represent the middle layer weights connected to the first neuron.
- *w*_21_^1^, *w*_22_^1^,…, *w*_2*n*_^1^ represent the middle layer weights connected to the second neuron.
- *w*_*q*1_^1^, *w*_*q*2_^1^,…, *w*_*qn*_^1^ represent the middle layer weights connected to neuron *q*. *q* here denotes the number of neurons in this layer.
- The weights associated with the output and neurons of the middle layer are denoted by *w*_21_, *w*_22_,…, *w*_2*q*_.

Now, the output of this neural network is gained through the steps described below:

(1) Neural network input, control signal, and system output in the previous sample times.
(2) The neuron's output in the middle layer is calculated based on the equations below:
  (10) $$\begin{array}{c} o_{i}=g\left(net_{i}\right),i=1,\ldots ,q,\\ net_{i}=w_{i}^{1}U, \end{array}$$
where
  (11) $$\begin{array}{c} U={\left[u\left(t-\tau_{1}\right),u\left(t-\tau_{2}\right)\ldots ,u\left(t-\tau_{n}\right)\right]}^{T},\\ w_{i}^{1}=\left[w_{i1}^{1},w_{i2}^{1},\ldots ,w_{in}^{1}\right],\\ g\left(net_{i}\right)=\frac{1-\exp\left(-net_{i}\right)}{1+\exp\left(-net_{i}\right)}. \end{array}$$

The MLP neural network's output can be calculated using the equation below:

(12) $$\begin{array}{c} y=w_{2}O, \end{array}$$

where

(13) $$\begin{array}{c} O={\left[o_{1},o_{2},\ldots ,o_{q}\right]}^{T},\\ w_{2}=\left[w_{21},w_{22},\ldots ,w_{2q}\right]. \end{array}$$

The weights of this neural network are trained in line with minimizing the cost function of *E*:

(14) $$\begin{array}{c} E=\frac{1}{2}e_{est}^{2}=\frac{1}{2}{\left(y_{d}-\hat{y}\right)}^{2}. \end{array}$$

According to (14), *y*_*d*_ denotes the desired output, and $\hat{y}$ is the neural network's output. The relationship for weights at *t* + 1 is represented by *w*(*t* + 1) = *w*(*t*) − *η∂E*/*∂w*. Also, gradient descent and error back-propagation algorithm are employed in training. To obtain *∂E*/*∂w*, the chain differentiation rule of $\partial E/\partial w=\left(\partial E/\partial e\right)\left(\partial e/\partial \hat{y}\right)\left(\partial \hat{y}/\partial w\right)$ applies. According to (15), the rule of training weights is calculated by substituting $\partial E/\partial e=e,\partial e/\partial \hat{y}=-1,\partial \hat{y}/\partial w=0$. The equation below indicates the rule of training weights.

(15) $$\begin{array}{c} w_{2}\left(t+1\right)=w_{2}\left(t\right)+\eta e_{est}O. \end{array}$$

The adaptive rule for the weights of the first layer is obtained as follows:

(16) $$\begin{array}{c} w_{i}^{1}\left(t+1\right)=w_{i}^{1}\left(t\right)+\eta e_{est}g^{\prime }\left(net_{i}\right)w_{2i}U. \end{array}$$

Concerning (16), *w*_*i*_^1^ denotes the vector of the neurons' weights in the middle layer connected to the *i*-th layer. *g*′(*net*_*i*_) is the derivation of *g*(*net*_*i*_) based on *net*_*i*_ input. It is noteworthy that *η* represents the training rate of gradient descent, according to which the adaptive rate is considered constant.

### 4.2. Jacobian of the MLP and Type-2 Neural-Fuzzy Controller Structure

Using the obtained model, the system's Jacobian is calculated:

(17) $$\begin{array}{c} \frac{\partial \Delta \;f}{\partial u_{c}}=\left(\left[w_{11}^{1},w_{21}^{1},\ldots ,w_{q1}^{1}\right]\mathrm{diag}\left[g^{\prime }\left(net_{1}\right),\ldots ,g^{\prime }\left(net_{q}\right)\right]w_{2}\right). \end{array}$$

According to (17), *∂*Δ  *f*/*∂u*_*c*_ represents the derivative of system output relative to control input. The expression [*w*_11_^1^, *w*_21_^1^,…, *w*_*q*1_^1^] is the considered vector of weights connected to the first input and neurons of the middle layer. diag(*A*) denotes the diagonal vector of matrix *A*. The expression [*g*′(*net*_1_),…, *g*′(*net*_*q*_)] This vector represents the derivative of neurons' output based on their inputs in the middle layer. The vector of weights connected to output and neurons of the middle layer are represented by *w*_2_.

**Figure 3:**
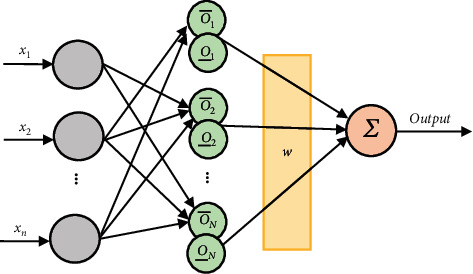
The structural definition for the type-2 neural-fuzzy controller.

Figure 3 illustrates the structure of the neural-fuzzy network. To clarify this type of network structure, giving attention to this figure is important.

According to Figure 3, *N* represents the number of middle layer neurons. Also, the displacement and the displacement derivative denote the number of neural-fuzzy network inputs. According to the fuzzy firing rules (18) the feedforward output of the controller is calculated through the equation below:

(18) $$\begin{array}{c} {\bar{O}}_{k}=\exp\left(-\frac{X-C_{k}^{2}}{{\bar{\sigma }}_{k}^{2}}\right),\\ {\underline{O}}_{K}=\exp\left(-\frac{X-C_{k}^{2}}{{\underline{\sigma }}_{k}^{2}}\right), \end{array}$$

where *C*_*k*_ is the center of the Gaussian function, *σ*_*k*_ is the width of the Gaussian function, and *k*=1,2,…, *N*.

Based on the order reduction of Nie-Tan, (19) is used to calculate the output of the neural-fuzzy network:

(19) $$\begin{array}{c} u_{C}=w^{T}Z. \end{array}$$

Concerning this equation, *w* is the vector of weights in the output layer, and *Z* is defined below:

(20) $$\begin{array}{c} Z={\left[z_{1},z_{2},\ldots z_{i},\ldots ,z_{N}\right]}^{T},\\ z_{i}=\frac{\left({\bar{O}}_{i}+{\underline{O}}_{i}\right)}{\sum_{i=1}^{N}\left({\bar{O}}_{i}+{\underline{O}}_{i}\right)}. \end{array}$$

Concerning (20), *N* denotes the number of rules or neurons in the middle layer.

### 4.3. Considering Error Back-Propagation and Extended Kalman Filter to Train Type-2 Neural-Fuzzy Controller

The weights training according to the error back-propagation and the extended Kalman filter are presented here. In the beginning, the square of the instantaneous error between the optimal response (the roof displacement becomes zero) and the network output at *t* is known as a cost function:

(21) $$\begin{array}{c} E=\frac{1}{2}e^{2}=\frac{1}{2}{\left(\Delta f\right)}^{2}. \end{array}$$

Concerning (21), *e* denotes the roof displacement error in the *x* axial. Due to the training rule of the error back-propagation and the extended Kalman filter, the cost function is derived from the neural-fuzzy network parameters.

(22) $$\begin{array}{c} w\left(t\right)=w\left(t-1\right)+p\left(t\right)\varphi \left(t\right)e\left(t\right),\\ p\left(t\right)=p\left(t-1\right)\left[I-K\left(t\right)\varphi \left(t\right)\right]+Q_{p}\left(t\right),\\ K\left(t\right)=\frac{p\left(t-1\right)\varphi^{T}\left(t\right)}{R_{m}\left(t\right)+\varphi \left(t\right)p\left(t-1\right)\varphi^{T}\left(t\right)}, \end{array}$$

where *∂E*/*∂w* denotes the cost function according to the neural-fuzzy network parameters and is calculated through (23), *w* depicts the weights vector in the last subsections. *φ*(*t*) represents the derivative output of the neural-fuzzy system relative to the parameters of the rules. Using a chain derivative, *∂E*/*∂w* is obtained as follows:

(23) $$\begin{array}{c} \frac{\partial E}{\partial w}=\frac{\partial E}{\partial \Delta \;f}\frac{\partial \Delta \;f}{\partial u_{c}}\frac{\partial u_{c}}{\partial \hat{y}}\frac{\partial \hat{y}}{\partial w}, \end{array}$$

where *u*_*c*_ denotes the control signal, and the output in the MLP neural network is represented by $\hat{y}$. Notably, *∂*Δ  *f*/*∂u*_*c*_ is the system's Jacobian, which was obtained through the neural system model and is calculated as follows:

(24) $$\begin{array}{c} E=\frac{1}{2}e^{2}=\frac{1}{2}{\left(\Delta f\right)}^{2}\Rightarrow \frac{\partial E}{\partial \Delta \;f}=\Delta f,\\ u_{c}=\hat{y}\Rightarrow \frac{\partial u_{c}}{\partial \hat{y}}=1,\\ \hat{y}=w^{T}Z\Rightarrow \frac{\partial \hat{y}}{\partial w}=Z, \end{array}$$

where *Z* is obtained through equation (20).

**Figure 4:**
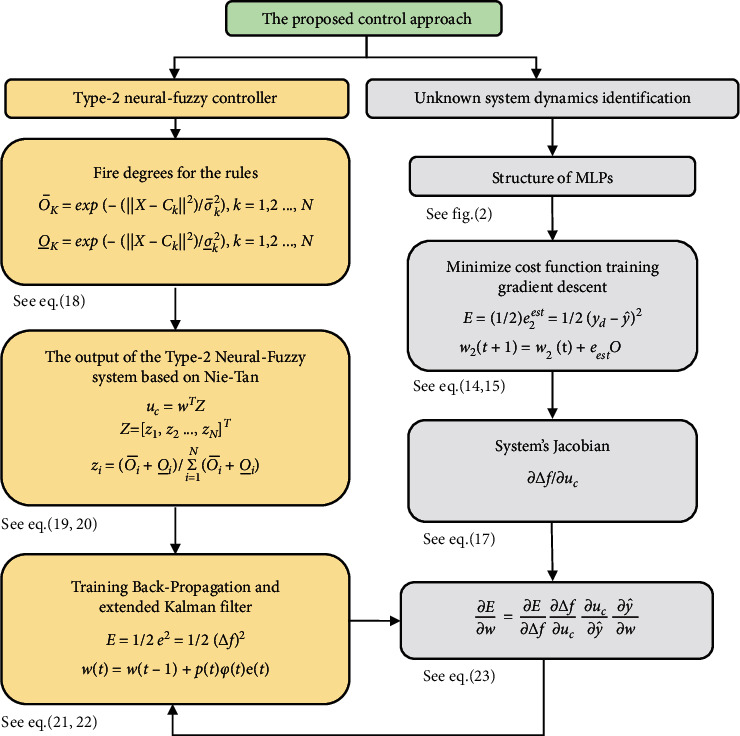
The flowchart of the structural control strategy according to the proposed approach.

The flowchart of the method proposed in this paper is shown in Figure 4.

### 4.4. Structure of the Proposed Controller

Structural systems have nonlinear dynamics as well as parametric and seismic uncertainties. Under such conditions, controllers with fixed interest rates do not work correctly. Hence, an adaptive or robust controller is necessary to tackle these uncertainties. On the other hand, a trade-off needs to be created between stability and accuracy to make an ideal controller work properly. It is noteworthy that many studies have considered the approaches of intelligent control, such as fuzzy logic, artificial neural networks (ANN), or neural-fuzzy networks, to identify complex systems and construct advanced controllers so far [119–127].

These studies are often conducted in two methods, which are as follows:

- When the system dynamics are determined, the controller is designed online.
- When the controller is designed offline, the system's dynamics are unknown.

**Figure 5:**
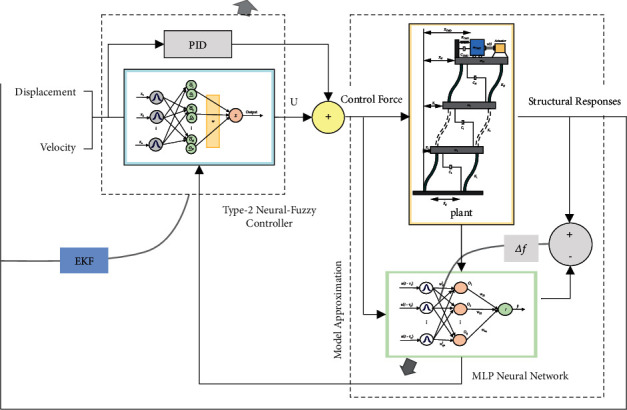
The Structure of the Proposed Control Strategy.

For this purpose, in the second case, evolutionary algorithms are employed to optimize the controller, and the optimized parameters are applied to the system. The basis of the assumption considered here is that the system parameters are indirectly known. It is worth mentioning that the main drawback of these methods is that they are time-consuming, and an optimal operating point may not be achieved. In this case, the computational work is expected to rise, leading to system instability. However, in both methods, the controllers are not resistant to uncertainties and cannot overcome parameter changes. Therefore, a novel adaptive type-2 neural-fuzzy controller (AT2NF) is considered here that, assuming the system dynamics are unknown, it can model more uncertainty than type 1 fuzzy systems. The proposed controller strikingly reduces the sensitivity to system parameters and raises the output response speed. In addition, this controller optimizes the computations through order reduction and can adapt itself to new conditions. The proposed method block diagram is demonstrated in Figure 5. In this paper, the system parameters are presumed to be unknown in this method; however, the controller is designed online. In this case, the neural-fuzzy network output, which is the control signal, is obtained online. In addition, the parameters of this neural-fuzzy network are adjusted so that the roof displacement error tends to be minimized. Therefore, the considered control aim is achieved. Calculating the cost function is based on the gradient descent that needs the system's Jacobian. The system's Jacobian is unknown because the system dynamics are assumed to be unknown. Hence, the system's Jacobian is extracted online through modeling the system using the MLP neural network (see Figure 5).

The main qualities of this proposed control method are as follows:

- This adaptive type-2 neural-fuzzy controller is designed not to require initial settings by the operator.
- This controller can overcome uncertain parameters and a time-varying system.
- A PID controller has been added to the type-2 neural-fuzzy controller to increase stability and robustness.
- The dynamics of the structural system are considered entirely uncertain.
- In contrast to other similar methods, there is no need for the Jacobian of the plant.
- The performance of type-2 adaptive neural-fuzzy controllers in a larger scale structural system can be evaluated.

### 4.5. Alternative Controller

This paper uses a simple adaptive controller (SAC) to evaluate the Type-2 neural-fuzzy system controller. Sobel et al. introduced the SAC method based on classical implicit or direct reference adaptive control [2, 19]. Then, this algorithm was extended by Bar-Kana et al., which improved many problems of the classical method when running a multiple-input multiple-output (MIMO) system [128, 129].

**Figure 6:**
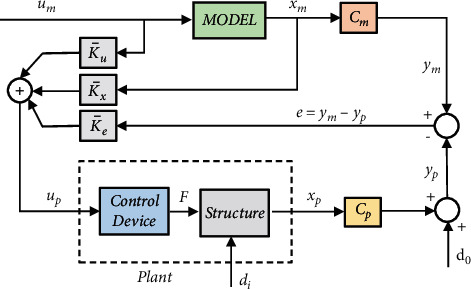
Simple adaptive control system block diagram [19].

It should be noted that the direct methods develop the adaptation process without explicit calculation of structural parameters. In other words, the SAC is not required to identify the controlled system's parameters fully, referred to as the plant, to obtain the necessary control gains for tracking arbitrary behavior in the control process. The primary aim of the SAC is to compel plant outputs to follow the ideal system's behavior, namely the reference model. Also, it allows the reference states to have less order than the plant [130]. The diagram block of SAC is indicated in Figure 6 [2].

The controlled structure's dynamic behavior (plant) in the form of state space is indicated as follows [128, 129] (see Figure 6):

(25) $$\begin{array}{c} {\overset{`}{x}}_{p}\left(t\right)=A_{p}x_{p}\left(t\right)+B_{p}u_{p}\left(t\right)+d_{i}\left(t\right),\\ y_{p}\left(t\right)=C_{p}x_{p}\left(t\right)+d_{o}\left(t\right), \end{array}$$

where *x*_*p*_ denotes the state vector of plan *n* × 1, *u*_*p*_ indicates the input control vector of *m* × 1, *y*_*p*_ represents the output plant of *q* × 1. Also, the state matrix of *n* × *n* is shown by *A*_*p*_, *B*_*p*_ is the input matrix of *n* × *m*, and *C*_*p*_ indicates the output matrix of *q* × *n*. According to the equation above, *d*_*i*_(*t*) is the disturbance to the system, and *d*_0_(*t*) is the disturbance in the sensors [129, 130]. The reference model in the state space is indicated as follows [129]:

(26) $$\begin{array}{c} {\overset{`}{x}}_{m}\left(t\right)=A_{m}x_{m}\left(t\right)+B_{m}u_{m}\left(t\right),\\ y_{m}\left(t\right)=C_{m}x_{m}\left(t\right), \end{array}$$

where *x*_*m*_ indicates the state vector of the reference model of *n*_*m*_ × 1, *u*_*m*_ shows the input control vector of *m* × 1, and *y*_*m*_ denotes the reference output vector of *q* × 1. Besides, the state matrix of *n*_*m*_ × *n*_*m*_ is indicated by *A*_*m*_, *B*_*m*_ denotes the input matrix of *n*_*m*_ × *m*, and the output matrix of *q* × *n*_*m*_ is shown by *C*_*m*_ [129, 130]. Plant *n* has a smaller order than the reference model *n*_*m*_. Note that this value must be sufficient to carry out the desired operation to create the plant [1, 111]. It is noteworthy that this ideal model (reference) demonstrates only the desired behavior (selected by the designers) and does not require that previous knowledge of the plant's dynamic parameters be provided [110]. SAC attempts to minimize (approaching zero asymptotically) the output tracking error (the output of the reference model and the output of the plant) denoted by *e*_*y*_.

The control commands must be calculated based on the whole available data for the ideal model by considering the states and inputs of the model in a feedforward configuration [128, 131].

(27) $$\begin{array}{c} e_{y}\left(t\right)=y_{m}\left(t\right)-y_{p}\left(t\right),\\ u_{p}\left(t\right)={\bar{K}}_{e}\left(t\right)e_{y}\left(t\right)+{\bar{K}}_{x}\left(t\right)x_{m}\left(t\right)+{\bar{K}}_{u}\left(t\right)u_{m}\left(t\right)=\bar{K}\left(t\right)\bar{r}\left(t\right), \end{array}$$

where

(28) $$\begin{array}{c} \bar{K}\left(t\right)=\left[\begin{array}{ccc} {\bar{K}}_{e}\left(t\right) & {\bar{K}}_{x}\left(t\right) & {\bar{K}}_{u}\left(t\right) \end{array}\right],\\ \bar{r}{\left(t\right)}^{T}={\left[\begin{array}{ccc} e_{y}\left(t\right) & x_{m}\left(t\right) & u_{m}\left(t\right) \end{array}\right]}^{T}. \end{array}$$

According to the equations above, the time-varying stabilizing control gain matrix is represented by ${\bar{K}}_{e}\left(t\right)$. It is worth mentioning that only the first expression in (27), namely, ${\bar{K}}_{e}\left(t\right)e_{y}\left(t\right)$ Is needed for the control system's stability. As a result, ${\bar{K}}_{x}\left(t\right)$ and ${\bar{K}}_{u}\left(t\right)$ denote the time-varying feedforward control gains required for obtaining zero output tracking error. These control gains are generated by the SAC method to ensure the stability of the controlled system and reduce the tracking error to zero asymptotically [109, 132]. When there is a disturbance, the coefficient of $\bar{r}{\left(t\right)}^{T}$ in (28) is used, and it might be quite small [133]. Adaptive control gains $\bar{K}\left(t\right)$ can be derived using a combination of integral and proportional terms [128, 131].

(29) $$\begin{array}{c} \bar{K}\left(t\right)=K_{I}\left(t\right)+K_{p}\left(t\right), \end{array}$$

where

(30) $$\begin{array}{c} {\dot{K}}_{I}\left(t\right)=e_{y}\left(t\right)\bar{r}{\left(t\right)}^{T}\bar{\Gamma_{I}}-\bar{\sigma }K_{I}\left(t\right), \end{array}$$

(31) $$\begin{array}{c} K_{p}\left(t\right)=e_{y}\left(t\right)\bar{r}{\left(t\right)}^{T}\bar{\Gamma_{p}}. \end{array}$$

According to (30), the positive-definite diagonal matrix is denoted by $\bar{\Gamma_{I}}$, which defines the control gains' rate of adaptation. Notably, the matrix of constant coefficients is represented by $\bar{\Gamma_{p}}$. Also, the proportional term ${\bar{K}}_{p}\left(t\right)$ is known as the immediate fine for large-scale errors [129, 131], which quickly directs the system towards small-scale errors. In addition, $\bar{\sigma }$ is a forgetting term matrix in the equation above, which is employed to prevent integral gain divergence under disturbances.

Regardless of the $\;\bar{\sigma }$ term, ${\bar{K}}_{I}\left(t\right)$ is a complete integrator and has the potential to grow indefinitely whenever full tracking (*e*_*y*_ = 0) is not possible. As a result, this term may reach unneeded and large values or even become divergent [129]. It should be noted that the integral adaptive control terms only guarantee the stability of the direct adaptive algorithm in equation (29). To increase the convergence of the closed-loop system towards complete tracking, the proportional adaptive control terms are added [128, 131]. To set the SAC controller, the parameters in equations (30) and (31) must be adjusted correctly. The process of selecting is often conducted by trial and error, requiring many sensitivity analyses, and there is doubt whether it leads to the most appropriate values or not [109, 132]. According to equation (32), the reference model is selected so that the output *y*_*m*_ is limited to $-{\bar{Y}}_{max}$ and ${\bar{Y}}_{\max}\left(-{\bar{Y}}_{\max}\leq y_{m}\leq {\bar{Y}}_{\max}\right)$ at any time under unknown inputs of *u*_*m*_. The acceleration of the earthquake is presumed to be not measured by any sensor. Hence, the term ${\bar{K}}_{u}\left(t\right)u_{m}\left(t\right)$ is eliminated from the procedure of generating the control command. The reference model states are considered as follows:

(32) $$\begin{array}{c} x_{m}=\left[\begin{array}{c} {\bar{q}}_{m}\\ {\dot{\bar{q}}}_{m} \end{array}\right]=\left[\begin{array}{c} \int {\dot{\bar{q}}}_{m}dt\\ {\dot{\bar{q}}}_{m} \end{array}\right]=\left[\begin{array}{c} \int y_{m}dt\\ y_{m} \end{array}\right],\\ y_{m}=y_{p}\;if\;\left|y_{p}\right|<{\bar{Y}}_{\max},\\ y_{m}=sign\left(y_{p}\right)y_{\max}\;if\;\left|y_{p}\right|\geq {\bar{Y}}_{\max}, \end{array}$$

where ${\bar{q}}_{m}$ and ${\dot{\bar{q}}}_{m}$ indicate the displacement and velocity of the reference model and ${\bar{Y}}_{\max}$ represents the vector of the maximum acceptable value for the model output. ${\bar{Y}}_{\max}$ can have any value equal to or greater than zero. The optimal value for *Y*_max_ depends on the purpose of the study, such as minimizing drift, acceleration, or other structural responses. According to this study, it is assumed that ${\bar{Y}}_{\max}=0$ causes the controller to reduce the displacement of the top story of the structure. It is novel that the parameters of the matrix $\bar{\sigma }$ and diagonal matrices $\bar{\Gamma_{I}}$ and $\bar{\Gamma_{p}}$ are adjusted at any time utilizing the extended Kalman filter. Accordingly, the OSAC needs to be presented as a novel controller.

## 5. Numerical Study

### 5.1. Structural Characterization and Dynamics

In the beginning, a structure with an 11-story realistic building is considered. This structure is situated in Rasht, one of Iran's cities. The purpose here is to numerically study and clarify the advantages of the proposed control strategy in minimizing the seismic response of the structure [48]. The story levels are assumed to have a rigid diaphragm, and the whole building mass is lumped at the story levels. Hence, a simplified linear model is considered for this structure. Also, the building containing the rigid beams and the columns with axially rigid and flexible features to lateral deformation is being considered. Thus, spring stiffness is established instead of the equivalent stiffness on each floor.

According to the assumptions that have been presented up to now, a shear-type building model could be a good choice to analyze the problem in this study. Accordingly, a degree of freedom (DOF) can define the displacements at each floor level. The top story contains a TMD, and in this case, one item is added to the DOF of the main structure. Therefore, the displacement of the structure stories and the TMD/ATMD systems is defined by 12 degrees of freedom. Since analyzing with the high expedition is generally desirable, a two-dimensional shear building can be considered. The control system employed here has an ATMD on the top story of the building. The stories' masses range from the first floor to the top are equal to 215, 201, 201, 200, 201, 201, 201, 203, 203, 203, and 176 tons. Furthermore, the values of 468, 476, 468, 450, 450, 450, 450, 437, 437, 437 and 312 MN/m are assigned to the related stiffness coefficients. TMD can be modeled on the highest floor using a linear spring and a viscous damper. The frequency ratio, namely, *β*_*TMD*_ is commonly presumed to be the ratio of the natural frequency of the TMD to the first modal frequency in the primary structure.

Furthermore, *α*_*TMD*_-percent of the total mass of the building is considered for the TMD mass, and *ξ*_*TM*  *D*_ -percent of the critical damping value is assigned to the damping ratio of the TMD. Using a genetic algorithm, the optimal values of *α*_*TMD*_, *ξ*_*TMD*_, and *β*_*TMD*_ are specified and turned out to be 3%, 7%, and 1.0, respectively. Then, the values of *ω*_1_ = 6.57 and *ω*_2_ = 19.36 rad/s are obtained for the uncontrolled structure's first and second natural frequencies. In addition, the structural damping ratio value is equal to 5% of the critical damping value in the first two modes. The supplementary information regarding this issue is given in the study of Pourzeynali et al. [48]. The damping matrix is computed using Rayleigh's method, shown in equation (5).

Table 2 illustrates the various values of Δ_*M*_ and Δ_*K*_that it is assumed that five structural models are considered to demonstrate the strength and stability of the proposed controller. Accordingly, ±15% and ±25% uncertainty are considered for the mass matrix and the structural system's stiffness coefficients, respectively.

**Figure 7:**
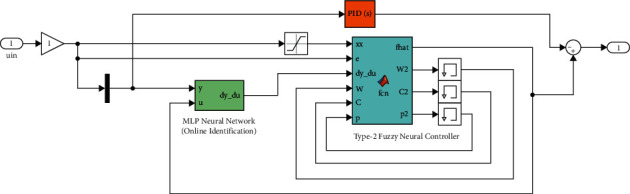
The block diagram for the proposed control strategy and the online identification.

A building simulation to perform time history analyses of both the uncontrolled and controlled (with TMD/ATMD system) structure is conducted employing MATLAB/Simulink.  Figure 7 outlines a block diagram of the SIMULINK software's online identification and control implementation [134].

**Figure 8:**
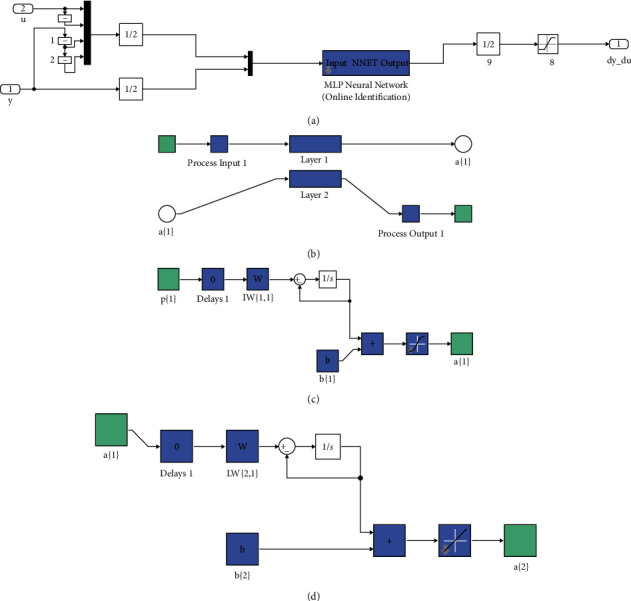
The inputs of the block diagram of the proposed control strategy.

The necessary information concerning this figure can be obtained from Figures 8(a)to 8(d). Accordingly, the content of Figure 8(a) is obtained by clicking on the green block in Figure 8. By clicking on the blue block of Figure 8(a), Figure 8(b) is obtained in which two layers exist. The contents of Layers 1 and 2 are respectively shown in Figures 8(c) and 8(d). Hence, the specific flowchart of the MLP Neural Network is more clearly outlined in Figure 8.

### 5.2. Earthquake Suite

**Figure 9:**
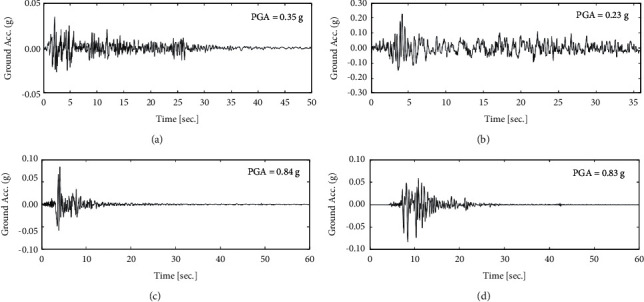
The time history of the considered earthquakes. (a) El Centro 1940, (b) Hachinohe 1968, (c) Northridge 1994, and (d) Kobe 1995.

To clarify the effectiveness of the proposed adaptive type-2 neural-fuzzy controller (AT2NF) implementation, the controlled structure with the ATMD system will be evaluated after being subjected to four specific earthquakes. It is worth mentioning that the International Association for Structural Control (IASC) reported two earthquakes far from the fault, the El Centro fault in 1940 and Hachinohe in 1968, and two earthquakes near the fault, the Northridge fault in 1994 and Kobe in 1995. The absolute maximum value for these earthquake records' ground acceleration (PGAs) equals 0.34g, 0.22g, 0.83g, and 0.82g, respectively.  Figure 9 outlines the time history of the four earthquakes based on the value of PGA.

## 6. Results and Discussion

This section discusses and assesses the outcomes of employing the proposed adaptive controllers, namely the OSAC and AT2NF Controller, in the 11-story structure equipped with the ATMD system. The proposed adaptive controllers' performance will be compared to several other controllers used in earlier studies. In addition, the intended uncertainties are taken into account when evaluating such controllers.

### 6.1. Examination and Comparison Results

To evaluate the OSAC and AT2NF controllers, analyses of the time histories of structural excitations under four well-known earthquakes are investigated. The results highlighted in Tables 3–6 are considered for making more comparisons between the proposed controllers and other control strategies presented in the previous studies. This is followed by a comparison of the maximum displacements of stories for the OSAC-controlled structure, the TMD-controlled structure [48], the FLC-controlled structure [48], the FOPID-controlled structure [68], and the OSMC-controlled structure [63] obtained under the El Centro, Hachinohe, Kobe, and Northridge earthquake excitations. The fuzzy logic controller of reference [48] is designed based on two input variables, namely displacement and velocity of the top floor of the structure. An input variable contains five trapezoidal membership functions, while an output variable contains seven triangular membership functions as an active external control force. A fuzzy associative memory (FAM) maps FLC input variables onto output variables. A genetic algorithm is used to optimize the membership function and weighting factors of the FAM (see Tables 3to 6).

The results indicate that TMD as a passive device can reduce the structure's seismic responses, but its performance depends on the seismic inputs. The results obtained for the Northridge earthquake show the lowest reduction, while the highest reduction is related to the El Centro earthquake. The numerical results emphasize the remarkable capabilities of ATMD as an active control in controlling the seismic responses of the four earthquakes with different frequency content. The numerical results indicate the unique advantages of OSAC over the previous controllers, including LQR, FLC, OSMC, and FOPID, in terms of mitigating and controlling the displacements of the structure. FLC, which generally outperforms LQR, has reduced the maximum top story displacement of the structure by about 0.051, 0.057, 0.37, and 0.17 m in the El Centro, Hachinohe, Kobe, and Northridge earthquakes, respectively. The performance of FOPID is close to the OSAC, but it can never provide the maximum reduction of the displacement same as OSAC. The OSAC and AT2NF controllers perform acceptable performance in near-fault and far-fault earthquakes. Nevertheless, the superiority of the AT2NF controller compared to the OSAC is considerable for the four earthquake excitations because, in this control method, the dynamics of the structural system are considered entirely uncertain, and this controller can overcome the uncertain parameters and the time-varying system and provide a more accurate estimate of the condition of the structure. AT2NF controller reduces about 87.3%, 86.4%, 80.6%, and 84.7% in the maximum structural responses for the mentioned earthquake excitations. In contrast, such values are 76.3%, 73.4%, 70.1%, and 69.4% for the OSAC under the El Centro, Hachinohe, Kobe, and Northridge earthquakes. The root mean squared (RMS) values of the structure's top stories displacement under the El Centro earthquake are approximately 3.01, 1.46, and 0.57 cm for the uncontrolled, OSAC, and AT2NF controllers, respectively. Thus, the OSAC and AT2NF controllers result in a reduction of approximately 51% and 81%, respectively, in comparison to the uncontrolled case. These reductions for the OSAC are about 60%, 62%, and 54% during the Hachinohe, Northridge, and Kobe earthquakes, respectively. Similarly, these reductions are approximately 86%, 82%, and 79% for the AT2NF controller.

**Figure 10:**
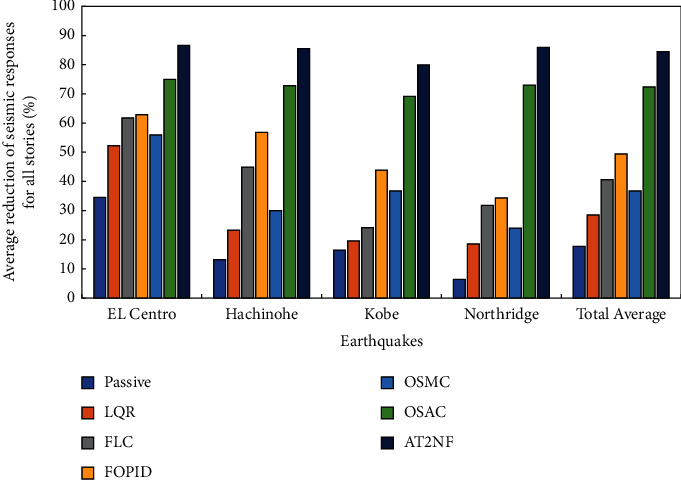
The average reduction of maximum structural responses for all stories.

It is concluded that the AT2NF controller outperforms the OSAC to reduce the structure's maximum and RMS seismic reactions. It is noteworthy that the AT2NF controller and OSAC generally demand the upper limit of the permissible control force, which is typically 5% total weight of the structure for the whole seismic excitations. As stated in the introduction, the control algorithm plays a prominent role in tuning the control force. Since the AT2NF controller does not depend on the structural parameters and has a remarkable capability in identification, it managed to provide a more accurate estimation regarding the structure condition and apply the control force to the structure at the right time. All these benefits of the AT2NF controller are the main reasons why the performance of this controller in the maximum reductions is better than the OSAC. In Figure 10, the averaged reductions in displacement for all stories for different earthquake excitations to demonstrate TMD's overall performance are illustrated. This figure also displays the total average of the reductions in displacements for all stories and all earthquake excitations for TMDs and various controllers. While TMDs perform well in a narrow range of load disturbances, they decrease performance during some earthquakes, such as the Northridge and Kobe earthquakes. The control system of ATMD considerably increases the effectiveness of TMD for a wide range of earthquakes with varying intensities and frequencies; however, the control algorithm used plays a crucial role in the idea's efficacy. The performance of the OSAC and AT2NF controllers is considerably better than the rest in this case. Nevertheless, the AT2NF controller offers the best performance for reducing the maximum displacement of structural stories compared with the OSAC and other control strategies. Based on the total average reduction of the maximum displacement for all stories shown in Figure 10, the total average reduction for the AT2NF controller is 84.6%, which is about 12.2% better than the OSAC. Also, the FOPID controller, with a total average reduction of 49.5%, has the best performance after the AT2NF controller and OSAC. In contrast, this value for the FLC, OSMC, LQR, and passive controllers are equal to 40.6%, 36.7%, 28.5%, and 17.7%, respectively (see Figure 10).

**Figure 11:**
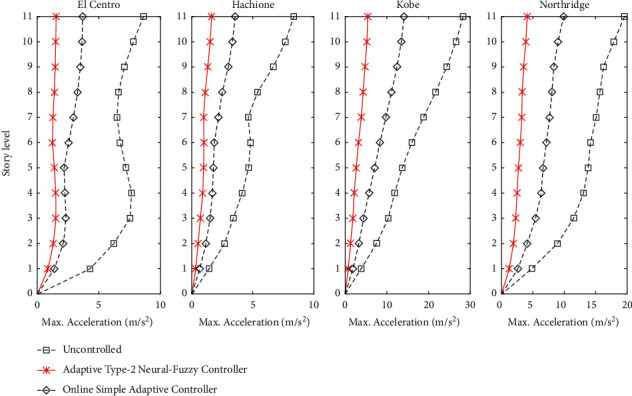
A comparison between the performance of proposed controllers and uncontrolled structure in terms of maximum acceleration.

According to Figure 11, the performance of the OSAC and AT2NF controller, along with uncontrolled structure, is evaluated in terms of the maximum acceleration of structural stories. In this case, the diagrams of the controller's performance are plotted based on the story level and maximum acceleration of structural stories.

In accordance with the numerical results presented in Table 7, the AT2NF controller has the remarkable capability of controlling the maximum acceleration of the structural stories. Notably, the AT2NF controller can, on a total average, reduce the maximum response by about 27% more than the OSAC.

**Figure 12:**
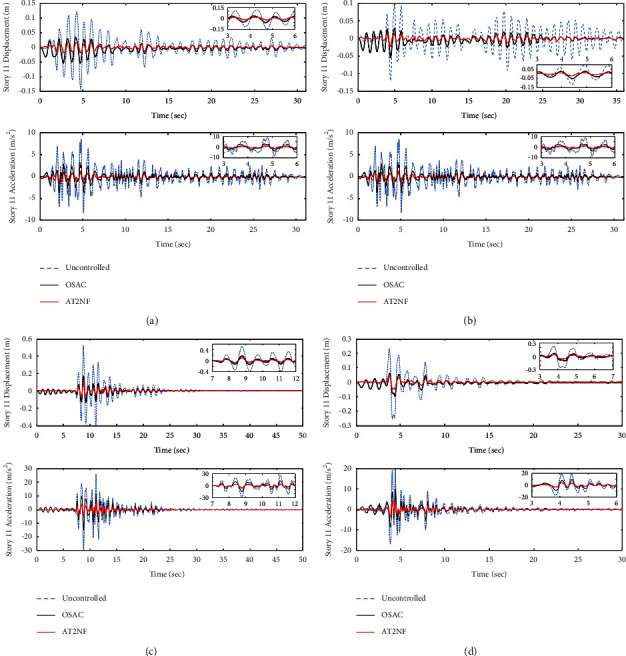
The performance of the proposed adaptive controllers in comparison with uncontrolled structure in terms of story 11 displacement and acceleration: (a) El Centro, (b) Hachinohe, (c) Kobe, and (d) Northridge.

Figure 12 compares the time histories of top story displacements and accelerations of the uncontrolled structure and the structure controlled by the ATMD control system with the proposed OSAC and AT2NF controllers. However, the AT2NF controller has a better performance in terms of reduction of maximum displacement and acceleration of structure under various earthquake excitation compared to the OSAC.

**Figure 13:**
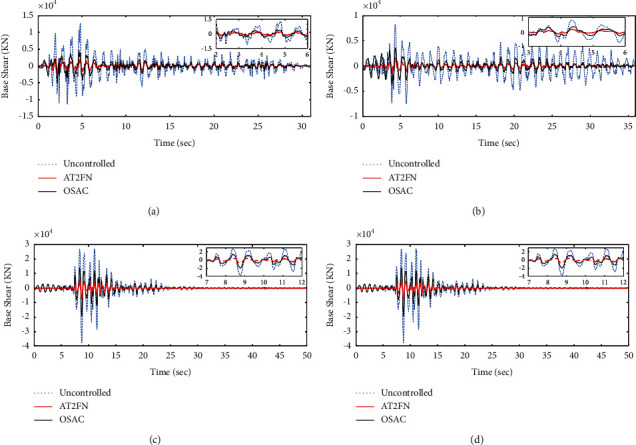
The performance of the proposed adaptive controllers in comparison with uncontrolled structure in terms of base shear. (a) El Centro, (b) Hachinohe, (c) Kobe, and (d) Northridge.

Figure 13 demonstrates the values of the base shear of the structure under the El Centro, Hachinohe, Kobe, and Northridge earthquakes. Accordingly, both proposed controllers have been successful in base shear reduction. However, since the value of base shear reduction for the AT2NF controller and OSAC is 81% and 54% on total average, the performance of AT2NF controller is considerably better.

### 6.2. Uncertainty Analysis

To assess the robustness of the online SAC controller and adaptive type-2 neural-fuzzy controller, a perturbation in the structural system is defined by −20% uncertainty in the initial stiffness matrix of the structure.

**Figure 14:**
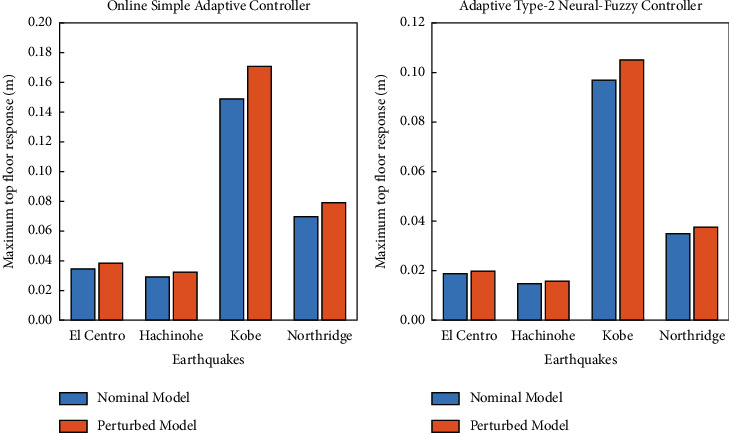
The maximum displacement response of the top floor of the structure of the nominal and perturbed model for the OSAC and AT2NF controller.

As shown in Figure 14, the maximum displacement response of the top floor of the structure of the perturbed model is compared with those for the nominal model for both controllers. According to the obtained results, the performance of both OSAC and AT2NF controllers is suitable against stiffness changes and can provide robust performance against stiffness uncertainties. Although, the performance of the AT2NF controller in tackling the uncertainties outperforms the OSAC, hence the deviation percentage from the nominal model for the OSAC under perturbation conditions is 10.3%, 11.7%, 14.8%, and 13.2% based on the El Centro earthquake, Hachinohe earthquake, Kobe earthquake, and Northridge earthquake, while the AT2NF controller has delivered more stable results with the deviation percentages of 5.3%, 6.3%, 8.2%, and 7.7% in such seismic excitations.

**Figure 15:**
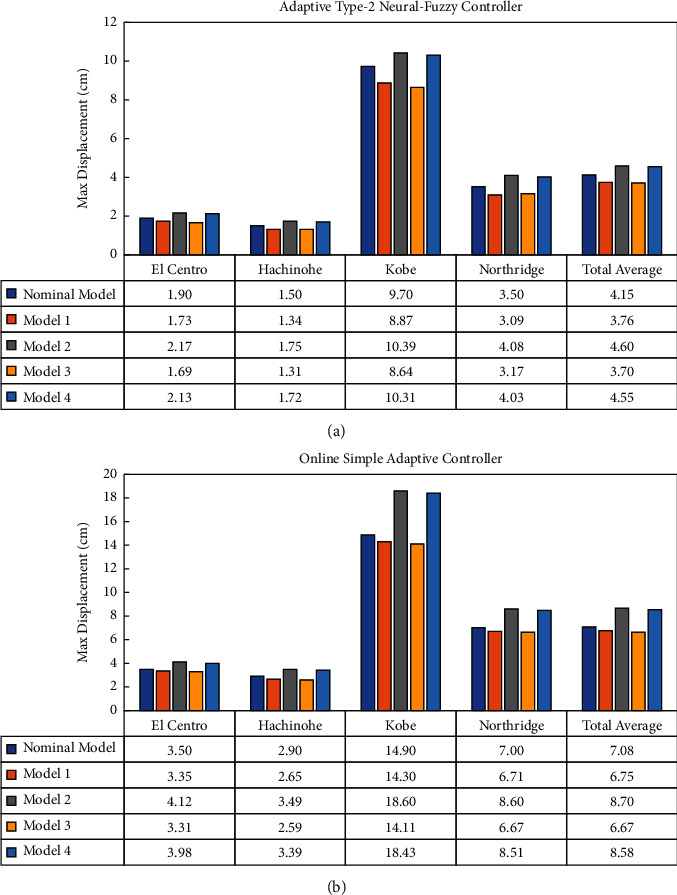
Examining the performance of (a) the AT2NF controller and (b) OSAC in terms of maximum displacement.

**Figure 16:**
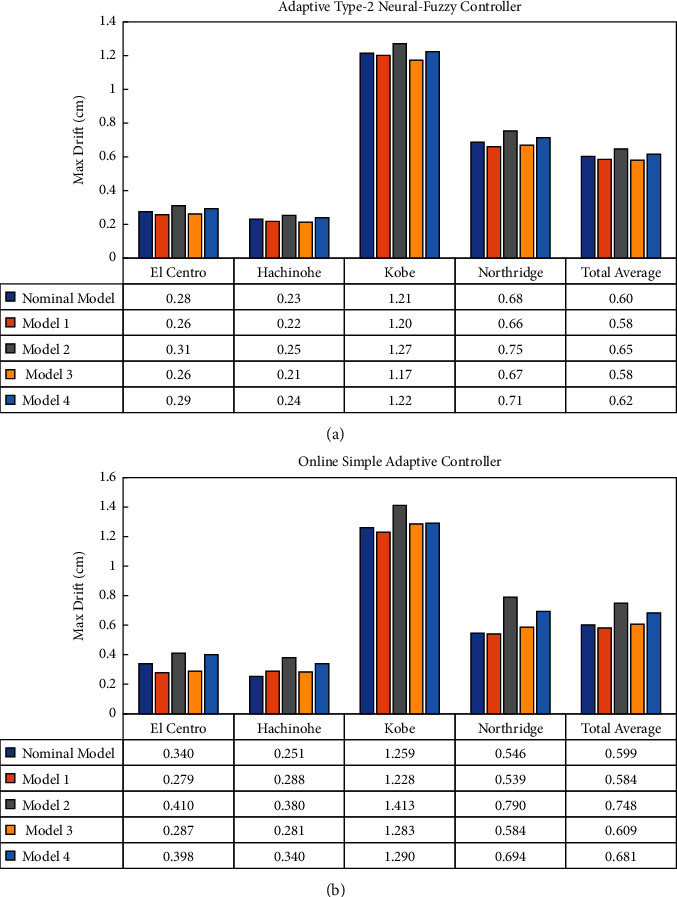
Examining the performance of (a) the AT2NF controller and (b) OSAC in terms of maximum drift.

Finally, a careful comparison is made between the AT2NF controller and the OSAC in terms of maximum displacement and maximum drift, as shown respectively in Figures 15 and 16.

As regards Figures 15 and 16, the robustness and stability of the proposed control methods under systematic parametric variations, as shown in Table 2, are assessed when controlling a seismically excited system equipped with ATMD. Accordingly, the control system's performance is expected to deteriorate when the parameters change. The obtained results imply that, in contrast to OSAC, the AT2NF controller did not lose its desirable performance under parametric changes. For instance, the deviation amount relative to the nominal model (AT2NF controller) in terms of maximum displacement for Model 2 is about 11% on total average, while this amount is about 23% for OSAC. According to the maximum drift for Model 2, the average deviation compared to the nominal model than all seismic records in AT2NF and OSAC controllers is approximately 7.5% and 25%, respectively. Hence, the OSAC seems to be more susceptible to parametric variation and does not maintain the general performance satisfactorily. Overall, it can be concluded that the AT2NF controller significantly outperforms the OSAC in terms of overcoming the parametric uncertainties.

## 7. Conclusion

The present paper proposes two robust adaptive controllers, namely, the AT2FN controller and the OSAC, to adjust the control forces of the ATMD-equipped structure on the top story of the structure. In the AT2NF controller, using the MLP, the Jacobian of the system is extracted, and the structural system model is estimated. Also, the controller parameters employed to adjust the control force applied to the ATMD were trained considering the extended Kalman filter (EKF) and the error back-propagation algorithm. To improve the system's stability and robustness against seismic vibrations, a PID controller was added to the adaptive type-2 neural-fuzzy controller. A careful comparison is made between the AT2NF controller and OSAC, which is based on the implicit reference model adaptive control and does not require complete identification of the controlled system parameters for obtaining the control gains needed to track the desired behavior in the control process. In this comparison, both controllers' ability to reduce maximum displacement, acceleration, base shear, and tackling the parametric uncertainties under far-field and near-field seismic excitations was examined. According to the obtained results, the AT2NF controller has significantly decreased the maximum structural responses under El Centro, Hachinohe, Kobe, and Northridge earthquakes and provides better performance than the OSAC. The major conclusions that were obtained in this paper are as follows:

- The AT2NF controller has been more successful than OSAC in maximum displacement reduction by 11.7%, 12.8%, 11%, and 13% on average under El Centro, Hachinohe, Kobe, and Northridge earthquake excitations, respectively.
- The AT2NF controller gives more acceleration reduction compared to OSAC by 27%.
- Regarding the base shear, the performance of the AT2NF controller is significantly better than OSAC, and these controllers have reduced the base shear on a total average of about 81% and 54%, respectively.
- It was revealed that the AT2NF controller has more robust than the OSAC against the parametric and seismic uncertainties.

Future studies could fruitfully explore this issue further by presenting better control methods. Further research should involve the adaptive form of learning rate *η*, which is now constant in the present study. Besides, to present a more efficient controller even better than the proposed AT2NF controller, we plan to consider the chaotic form of the membership functions in the future. In addition, an adaptive type-2 fuzzy PID controller can be added to the control system as a compensator to increase stability and robustness in the future. Furthermore, using an adaptive type-2 fuzzy sliding mode controller can be very beneficial.

## Figures and Tables

**Table 1:** A summary of the proposed controllers in the previous studies.

No	Controller	Targets	Type of structure	Features
1	Adaptive neural network control system [106]	Tackling the dynamic nonlinearities and uncertainties	A quarter car electrohydraulic active suspension system	The ability to tackle the unknown smoothing functions
2	An adaptive neural network control method [107]	Obtaining the precise and robust control of nonlinear systems with unknown dynamics	A generic single-input single-output nonlinear system with unknown dynamics	The network trained by an iterative control learning algorithm and a proportional-integral controller are combined in this controller
3	A novel online neural-network-based sliding mode control (OLNN-SMC) design [108]	Obtaining robust adaptive precision motions	Piezoelectric actuated (PEA) system	The ability to realize the nonlinearity of the PEA system using singularity-free neural networks (NNs)
4	Neural Network-Based Adaptive Controller [109]	Tackling the parametric uncertainties and external disturbances	Wheeled mobile robots	A neural network-based kinematic controller and a model reference adaptive control are combined
5	An adaptive neural network control strategy based on radial basis function neural network (RBFNN) [110]	Tracking control of the pneumatic servo system	pneumatic servo system	The proposed controller considers the state constraints to enhance the tracking accuracy
6	adaptive radial basis function (RBF) neural network-based active disturbance rejection controller (ADRC) [111]	Minimizing the effect of internal and external unknown uncertainties of the unmanned helicopter	An unmanned helicopter	Better anti-disturbance, robustness, and tracking accuracy compared to the traditional ADRC and PID approaches
7	An intelligent adaptive neural network (ANN) controller for Ref. [112]	Optimization of the parameters of a PI controller with real-time data and giving dynamic stability	A direct torque controlled (DTC) electric vehicle (EV) propulsion system	The stator reference flux voltage considered for synthesizing the space vector with width modulation is obtained for a DTC
8	Modified Simple Adaptive Control [113]	Examining the effect of aircraft weight on the controlled system response considering the various disturbing states	A simple adaptive shimmy suppression system	To avoid windup impacts, the saturation of actuator control moment and a simple back-calculation design are considered
9	An adaptive neuro-fuzzy inference system (ANFIS) and simple adaptive control (SAC) approaches [114]	Tackling the uncertainties of full three-dimensional models under multi excitations	Three-dimensional coupled buildings	The performance of both controllers was acceptable
10	A simple adaptive controller methodology and model predictive control (MPC) [115]	Creating and tracking trajectories of a spacecraft next to the asteroids	Spacecraft Near Asteroids	Adaptive control is used as a feedback controller and MPC as a feedforward controller for tackling the unknown uncertainties
11	A Simple Adaptive Control (SAC)-based reconfiguration approach [116]	Tackling the faults of sensors and actuators in the CPCS	Cabin Pressure Control System (CPCS)	The control method capability for controlling the rules online regardless of identifying the system under faults
12	Simple Adaptive Control (SAC) [117]	Reduction of the adverse effects of the earthquake on the structures	Six-story structure	The proposed controller has a striking performance under various seismic excitations.

**Table 2:** The uncertainty coefficients' values in the nominal and perturbed models.

Models	Nominal model	Model 1	Model 2	Model 3	Model 4
Δ_*M*_	0.00	+0.15	+0.15	−0.15	−0.15
Δ_*K*_	0.00	+0.25	−0.25	+0.25	−0.25

**Table 3:** A comparison of the performance of different controllers in terms of the maximum responses of stories during the El Centro earthquake.

Story	Maximum responses of stories (m)								Reduction amount based on the percentage (%)						
	Unctrl. [48]	Passive [48]	LQR [48]	FLC [48]	FOPID [68]	OSMC [63]	OSAC	AT2NF	Passive	LQR	FLC	FOPID	OSMC	OSAC	AT2NF
1	0.019	0.013	0.009	0.090	0.009	0.008	0.005	0.003	31.6	52.6	52.6	52.6	57.9	73.2	85.5
2	0.039	0.025	0.018	0.016	0.016	0.016	0.010	0.005	35.9	53.8	59.0	59.0	59.0	74.9	86.5
3	0.057	0.037	0.027	0.023	0.023	0.024	0.014	0.008	35.1	52.6	59.6	59.6	57.9	74.9	86.4
4	0.074	0.048	0.035	0.028	0.029	0.032	0.019	0.010	35.1	52.7	62.2	60.8	56.8	74.7	86.4
5	0.090	0.058	0.043	0.034	0.034	0.041	0.023	0.012	35.6	52.2	62.2	62.2	54.4	74.6	86.5
6	0.100	0.067	0.050	0.039	0.038	0.047	0.026	0.014	34.4	51.0	61.8	62.8	53.0	73.5	86.0
7	0.120	0.074	0.058	0.043	0.041	0.053	0.030	0.016	38.3	51.7	64.2	65.8	55.8	75.4	87.1
8	0.130	0.083	0.060	0.047	0.043	0.058	0.032	0.017	36.2	53.9	63.9	66.9	55.4	75.3	87.1
9	0.140	0.094	0.067	0.049	0.044	0.062	0.034	0.018	32.9	52.1	65.5	68.6	55.7	75.8	87.3
10	0.140	0.094	0.070	0.050	0.046	0.064	0.035	0.018	32.9	50.0	64.3	67.1	54.3	75.1	86.9
11	0.147	0.099	0.072	0.051	0.049	0.065	0.035	0.019	32.7	51.0	65.3	66.7	55.8	76.3	87.3
Average	0.096	0.063	0.046	0.035	0.034	0.043	0.024	0.0014	34.6	52.1	61.8	62.9	56.0	74.9	86.6

**Table 4:** Comparison of performance of the different controllers in terms of maximum responses of stories in the Hachinohe earthquake.

Story	Maximum responses of stories (m)								Reduction amount based on the percentage (%)						
	Unctrl. [48]	Passive [48]	LQR [48]	FLC [48]	FOPID [68]	OSMC [63]	OSAC	AT2NF	Passive	LQR	FLC	FOPID	OSMC	OSAC	AT2NF
1	0.014	0.012	0.011	0.008	0.007	0.011	0.004	0.002	14.3	21.4	42.9	50.0	21.4	73.1	83.6
2	0.028	0.024	0.021	0.017	0.005	0.021	0.007	0.004	14.3	25.0	39.3	82.1	25.0	73.9	84.3
3	0.040	0.035	0.032	0.024	0.005	0.030	0.011	0.006	12.5	20.0	40.0	87.5	25.0	73.0	84.3
4	0.053	0.046	0.041	0.030	0.005	0.039	0.014	0.008	13.2	22.6	43.4	90.6	26.4	73.2	85.0
5	0.064	0.055	0.050	0.036	0.051	0.047	0.018	0.009	14.1	21.9	43.8	20.3	26.6	72.6	85.4
6	0.074	0.064	0.058	0.040	0.050	0.052	0.021	0.011	13.5	21.6	45.9	32.4	29.7	72.1	85.8
7	0.085	0.073	0.065	0.046	0.047	0.057	0.024	0.012	14.1	23.5	45.9	44.7	32.9	72.2	86.4
8	0.094	0.081	0.071	0.050	0.042	0.062	0.026	0.013	13.8	24.5	46.8	55.3	34.0	72.2	86.6
9	0.100	0.089	0.076	0.053	0.046	0.066	0.028	0.014	11.0	24.0	47.0	54.0	34.0	72.0	86.4
10	0.110	0.095	0.079	0.055	0.050	0.068	0.029	0.014	13.6	28.2	50.0	54.5	38.2	73.6	86.9
11	0.110	0.099	0.083	0.057	0.051	0.070	0.029	0.015	10.0	24.5	48.2	53.6	36.4	73.4	86.4
Average	0.070	0.061	0.053	0.038	0.033	0.048	0.019	0.010	13.1	23.4	44.8	56.8	30	72.8	85.6

**Table 5:** A comparison of the maximum response times of different controllers to the Kobe earthquake.

Story	Maximum responses of stories (m)								Reduction amount based on the percentage (%)						
	Unctrl. [48]	Passive [48]	LQR [48]	FLC [48]	FOPID [68]	OSMC [63]	OSAC	AT2NF	Passive	LQR	FLC	FOPID	OSMC	OSAC	AT2NF
1	0.060	0.049	0.050	0.046	0.038	0.037	0.019	0.012	18.3	16.7	23.3	37.5	38.3	68.5	79.8
2	0.120	0.098	0.101	0.092	0.075	0.057	0.037	0.024	18.3	15.8	23.3	37.5	37.5	68.8	80.0
3	0.180	0.149	0.144	0.131	0.106	0.113	0.056	0.036	17.2	20.0	27.2	41.0	37.2	69.0	80.0
4	0.240	0.199	0.192	0.180	0.141	0.151	0.074	0.048	17.1	20.0	25.0	41.4	37.1	69.1	80.1
5	0.290	0.238	0.241	0.229	0.169	0.186	0.091	0.059	17.9	16.9	21.0	41.8	35.9	68.5	79.7
6	0.340	0.289	0.269	0.258	0.191	0.219	0.107	0.069	15.0	20.9	24.1	43.9	35.6	68.5	79.7
7	0.390	0.332	0.308	0.293	0.209	0.248	0.121	0.078	14.9	21.0	24.9	46.3	36.4	69.0	80.0
8	0.430	0.361	0.344	0.335	0.225	0.273	0.132	0.086	16.0	20.0	22.1	47.7	36.5	69.2	80.1
9	0.460	0.391	0.363	0.354	0.238	0.293	0.141	0.091	15.0	21.1	23.0	48.4	36.3	69.3	80.2
10	0.480	0.408	0.374	0.360	0.250	0.306	0.147	0.095	15.0	22.1	25.0	47.9	36.3	69.5	80.2
11	0.500	0.420	0.390	0.370	0.256	0.315	0.149	0.097	16.0	22.0	26.0	48.8	37.0	70.1	80.6
Average	0.317	0.267	0.252	0.241	0.173	0.201	0.098	0.063	16.4	19.7	24.1	43.8	36.7	69.0	80.0

**Table 6:** Comparison of the response times of different controllers during the Northridge earthquake.

Story	Maximum responses of stories (m)								Reduction amount based on the percentage (%)						
	Unctrl. [48]	Passive [48]	LQR [48]	FLC [48]	FOPID [68]	OSMC [63]	OSAC	AT2NF	Passive	LQR	FLC	FOPID	OSMC	OSAC	AT2NF
1	0.046	0.040	0.033	0.031	0.026	0.032	0.010	0.005	13.0	28.3	32.6	43.7	30.4	77.7	88.1
2	0.088	0.080	0.063	0.058	0.049	0.059	0.020	0.011	9.1	28.4	34.1	44.8	33.0	77.4	87.9
3	0.123	0.109	0.109	0.080	0.068	0.083	0.029	0.015	11.4	11.4	35.0	44.7	32.5	76.3	87.5
4	0.150	0.140	0.110	0.099	0.091	0.103	0.038	0.020	6.7	26.7	34.0	39.5	31.3	74.7	86.9
5	0.180	0.160	0.130	0.119	0.110	0.119	0.046	0.024	11.1	27.8	33.9	38.8	33.9	74.5	86.8
6	0.194	0.178	0.149	0.130	0.126	0.138	0.053	0.027	8.2	23.2	33.0	34.9	28.9	72.7	85.8
7	0.204	0.190	0.169	0.139	0.139	0.155	0.059	0.030	6.9	17.2	31.9	31.7	24.0	71.2	85.0
8	0.210	0.200	0.181	0.143	0.152	0.169	0.063	0.033	4.8	13.8	31.9	27.5	19.5	69.8	84.3
9	0.220	0.220	0.189	0.156	0.162	0.188	0.067	0.034	0.0	14.1	29.1	26.4	14.5	69.6	84.3
10	0.230	0.230	0.209	0.168	0.172	0.204	0.069	0.035	0.0	9.1	27.0	25.3	11.3	70.0	84.7
11	0.230	0.230	0.219	0.170	0.181	0.218	0.070	0.035	0.0	4.8	26.1	21.1	5.2	69.4	84.7
Average	0.170	0.162	0.142	0.118	0.116	0.133	0.048	0.025	6.5	18.6	31.7	34.4	24.1	73.0	86.0

**Table 7:** A comparison between the performance of proposed controllers and uncontrolled structure in terms of maximum acceleration.

The earthquake	Max responses in an absolute acceleration of stories (m/s^2^)			Percentage of reduction (%)	
	Unctrl.	OSAC	AT2NF	OSAC	AT2NF
El Centro	8.63	3.68	1.54	57	82
Hachinohe	8.35	3.55	1.62	57	81
Kobe	30.11	15.04	5.78	50	81
Northridge	19.57	9.86	4.07	50	79
Total Average	16.67	8.03	3.25	54	81

## Data Availability

Data are available and can be provided via email querying directly to the corresponding author (amousavi2000@iaut.ac.ir).
